# Isolation, Characterization, and Host-Range Evaluation of Bacteriophages Against Multidrug-Resistant Escherichia coli Clinical Isolates

**DOI:** 10.7759/cureus.117861

**Published:** 2026-10-13

**Authors:** Mukesh Kumar Singh, Ashutosh Rawat, Asim Kumar Singh

**Affiliations:** 1 Department of Microbiology, Santosh Medical College and Hospital, Santosh Deemed to be University, Ghaziabad, IND; 2 Department of Microbiology, Heritage Institute of Medical Sciences, Varanasi, IND

**Keywords:** antimicrobial resistance, bacteriophage, escherichia coli, host range, lytic activity, multidrug resistance, phage therapy

## Abstract

Background

The increasing prevalence of multidrug-resistant (MDR) *Escherichia coli *has created a need for alternative antibacterial approaches. Bacteriophages are being investigated because of their ability to specifically infect and lyse bacterial hosts. However, the host range of individual phages may be limited. The present study evaluated bacteriophages isolated from environmental water sources against clinical MDR *E. coli* isolates and assessed the observed lytic coverage of a three-phage cocktail.

Methods

An in vitro experimental study was conducted using 375 MDR *E. coli* clinical isolates recovered from clinical specimens. Pond water, sewage water, and Ganga water were screened for bacteriophages using a spot assay, followed by plaque assay and plaque purification. Purified phages were propagated, and their titers were determined by plaque assay. The host-range activity of three purified phages was evaluated against all 375 MDR *E. coli* isolates. The cumulative coverage of a three-phage combination was then derived from the individual host-range results. Lytic activity among the three individual phages was compared using Cochran's Q test, followed by exact McNemar tests with Bonferroni correction.

Results

Among the 375 MDR *E. coli* isolates, the highest resistance was observed against ciprofloxacin (95.2%), while the highest susceptibility was observed to amikacin (72.3%). Preliminary spot screening identified phage-associated lysis in 72 (19.2%) isolates, while plaque formation was observed in 36 (9.6%) isolates. Three purified bacteriophages were recovered from pond water, sewage water, and Ganga water and designated Phage-1, Phage-2, and Phage-3, respectively. Their estimated titers were 1.6 × 10¹², 1.7 × 10¹⁶, and 1.9 × 10¹⁵ PFU/mL, respectively. Phage-1 produced detectable lysis in 146 (38.9%) isolates, Phage-2 in 90 (24.0%), and Phage-3 in 169 (45.1%). Cochran's Q test demonstrated a significant overall difference in lytic activity among the three phages (p < 0.001). The difference between Phage-1 and Phage-3 was not statistically significant (p = 0.259). The cumulative coverage of the three phages combined, derived from the individual host-range results, was 260 (69.3%) isolates, while 115 (30.7%) showed no detectable lysis with any of the three phages.

Conclusion

The study demonstrated that environmental water sources yielded bacteriophages with detectable lytic activity against clinical MDR *E. coli* isolates. The individual phages showed variable host-range activity, and their combined (cumulative) coverage, derived from the individual host-range results, was broader than that of any individual phage. This represents cumulative phenotypic coverage and should not be interpreted as the result of a separately tested cocktail or as evidence of phage synergy. Further biological, genomic, safety, and efficacy studies are required to evaluate the potential of these bacteriophages for subsequent phage-development research.

## Introduction

Antimicrobial resistance (AMR) is a major global public health challenge that threatens the effective treatment of bacterial infections. The global burden of bacterial AMR has increased substantially over recent decades, with resistant Gram-negative bacteria representing an important component of this burden. The continuing emergence and dissemination of multidrug-resistant (MDR) organisms have reduced the effectiveness of conventional antimicrobial therapy and have increased the need to investigate alternative or complementary antibacterial strategies [1].

*Escherichia coli *is an important human pathogen and a major cause of UTIs, while resistant strains are also associated with other clinical infections. The increasing prevalence of antimicrobial-resistant *E. coli*, particularly among clinical isolates, presents a significant therapeutic challenge. Vera-Mansilla et al. demonstrated that lytic bacteriophages could be isolated and characterized against MDR clinical *E. coli*, supporting the feasibility of investigating phages as potential antibacterial agents against resistant clinical isolates [2].

Bacteriophages, or phages, are viruses that infect bacteria and use bacterial cells for their replication. Lytic bacteriophages can cause destruction of susceptible bacterial cells following productive infection and have therefore attracted renewed interest as potential antibacterial agents in the context of increasing AMR. Recent studies and reviews have highlighted the potential of bacteriophage-based approaches against antimicrobial-resistant bacterial infections while also emphasizing important biological and translational challenges [3].

An important limitation of bacteriophage-based antibacterial approaches is the variable host range of individual phages. Phage susceptibility can differ substantially between isolates of the same bacterial species. Successful infection depends on whether the phage can recognise and attach to the surface structures present on a given isolate and on whether that isolate carries defence mechanisms capable of blocking infection once attachment has occurred. Maffei et al., through systematic investigation of *E. coli* phage-host interactions using the BASEL phage collection, demonstrated considerable diversity in phage-host interactions and susceptibility patterns among bacterial isolates [4]. These observations emphasize the importance of experimentally determining the host range of candidate phages against relevant clinical bacterial populations.

The limited or variable host range of individual phages provides a rationale for investigating combinations of phages. Phage cocktails contain two or more bacteriophages selected to provide complementary or overlapping antibacterial activity and may increase the proportion of bacterial isolates susceptible to phage-mediated lysis. Experimental studies involving *E. coli* have demonstrated that combinations of phages can produce broader observed bacterial coverage than individual phages under the conditions tested [5]. However, increased coverage should not automatically be interpreted as evidence of synergistic interaction. Demonstration of synergy requires dedicated experimental evaluation using an appropriate quantitative assay.

Systematic characterization of candidate phages is therefore important before their consideration for subsequent therapeutic or translational research. Such characterization, including assessment of host range, growth properties, and activity against contemporary bacterial isolates, can facilitate the identification of phages with suitable lytic activity and provide a basis for selecting multiple phages for combination approaches [6].

Environmental sources represent important reservoirs for bacteriophages because phages are widely distributed in environments containing their bacterial hosts. Water-associated environments, including rivers, ponds, sewage, and wastewater, have been investigated as sources of bacteriophages active against clinically important bacterial species. The successful isolation of lytic phages against MDR clinical *E. coli* from environmental sources further supports environmental sampling as a practical approach for identifying phages with activity against resistant bacterial isolates [2].

The use of multiple phages against MDR *E. coli *has also been investigated experimentally. Korf et al. evaluated a phage cocktail against E. coli and demonstrated that combining phages could provide broader antibacterial activity than individual phages under the experimental conditions used [5]. Similarly, Kim et al. reported that a phage cocktail targeting different phylogenetic groups inhibited a high proportion of antimicrobial-resistant *E. coli* isolates, illustrating how complementary host ranges can increase observed coverage [7]. These findings provide a biological rationale for evaluating phage combinations when individual phages show complementary susceptibility patterns. However, the extent of coverage depends on the specific phages, bacterial isolates, and experimental conditions used.

The primary objective of the present study was to isolate bacteriophages from environmental water sources and to determine the host range of each purified phage individually against a contemporary collection of MDR *E. coli *clinical isolates. The secondary objective was to determine the cumulative coverage achieved when the host ranges of the individual phages were considered together, in order to assess whether their susceptibility profiles were complementary. Throughout, cumulative phenotypic coverage is reported as distinct from phage synergy, which was not evaluated in this study.

## Materials and methods

Study area

The study was conducted as a collaborative research study between the Department of Microbiology, Santosh Medical College and Hospital, Santosh Deemed to be University, Ghaziabad, Uttar Pradesh, India, and the Department of Microbiology, Heritage Institute of Medical Sciences (HIMS), Varanasi, Uttar Pradesh, India. Clinical MDR *E. coli *isolates were obtained from clinical specimens received in the microbiology laboratory. Environmental water samples, including pond water, sewage water, and Ganga water, were collected for isolation of bacteriophages active against MDR *E. coli*.

Study period

The study was conducted from October 2024 to September 2026.

Study design and clinical isolates

The present investigation was an in vitro experimental study designed to isolate and evaluate bacteriophages active against MDR *E. coli* clinical isolates. A total of 375 MDR *E. coli* isolates recovered from various clinical specimens received in the microbiology laboratory during the study period were included in the study, irrespective of specimen type. The bacterial isolates were identified as *E. coli *using routine microbiological identification procedures.

Antimicrobial susceptibility testing

Antimicrobial susceptibility testing was performed using the Kirby-Bauer disc diffusion method [8]. A standardized suspension of each bacterial isolate was prepared and inoculated onto the Mueller-Hinton agar medium to produce a uniform bacterial lawn. Antibiotic discs were subsequently placed on the inoculated agar surface according to standard disc-diffusion procedures.

Following overnight incubation at 37°C, the inhibition zones were measured, and the isolates were categorized as susceptible (S), intermediate (I), or resistant (R) according to the zone diameter interpretive breakpoints for* Enterobacterales* specified in CLSI M100, 34th edition (2024) [9]. Isolates were classified as MDR when they were non-susceptible to at least one agent in three or more antimicrobial categories, as defined by Magiorakos et al. [10].

The antimicrobial agents tested included ampicillin, amoxicillin-clavulanic acid, piperacillin-tazobactam, cefuroxime, ceftriaxone, ceftazidime, cefoperazone-sulbactam, cefepime, ertapenem, imipenem, meropenem, amikacin, gentamicin, ciprofloxacin, ofloxacin, trimethoprim-sulfamethoxazole, and, among urinary isolates, norfloxacin, fosfomycin, and nitrofurantoin.

The antimicrobial susceptibility profile was used to characterize the resistance pattern of the MDR *E. coli* study population before bacteriophage screening and host-range evaluation.

Collection of environmental samples

Three environmental water sources were sampled for the recovery of coliphages: pond water and sewage water, both collected from sites near HIMS, Varanasi, and river Ganga water, collected from Shultankeshwar Ghat, Varanasi.

One sample was collected from each source. Approximately 500 mL of water was collected from each site in a sterile wide-mouth container, which was filled from below the water surface so as to avoid the surface film and floating debris. The containers were labelled, sealed, and transported to the laboratory in a sample transport box. The samples were processed immediately on arrival at the laboratory; in case of any delay, they were stored at 4-5°C until processing. Processing involved screening of bacteriophages capable of producing lysis of MDR *E. coli *clinical isolates.

Preliminary screening of bacteriophages by spot assay

The environmental samples were screened for bacteriophages using the spot assay [11] against the MDR *E. coli* clinical isolates. Screening was performed directly against the clinical isolates; a standard reference strain, such as *E. coli *ATCC 25922, was not used as a screening host.

Bacterial lawns of the test isolates were prepared on appropriate agar plates. Aliquots of the processed environmental phage preparations were applied to the bacterial lawns, and the plates were examined for visible zones of lysis. Isolates showing detectable zones of lysis were considered positive in the preliminary spot assay and were subjected to plaque-based confirmation. Among the 375 MDR *E. coli* isolates screened, 72 (19.2%) demonstrated a positive reaction in the preliminary spot assay.

Plaque assay and confirmation of phage activity

Spot-positive phage preparations were subjected to plaque assay using the double-layer agar technique [11]. The phage preparation was mixed with the susceptible *E. coli* host culture and appropriate molten overlay medium and subsequently overlaid onto the base agar. Following overnight incubation at 37°C, the plates were examined for discrete plaques. Plaque formation was considered evidence of productive phage activity under the experimental conditions. Plaque formation was observed in 36 (9.6%) of the 375 MDR E. coli isolates.

Plaque purification and propagation

Individual plaques obtained during plaque assay were selected and subjected to repeated plaque purification to obtain purified bacteriophage isolates. The purified phages were subsequently propagated using susceptible *E. coli *host cultures. The propagated phage preparations were used for titer determination and subsequent host-range experiments. Three purified bacteriophages were ultimately recovered and designated according to their environmental sources as follows: Phage-1 (P1), isolated from pond water; Phage-2 (P2), isolated from sewage water; and Phage-3 (P3), isolated from Ganga water.

Determination of phage titer

The titer of each purified bacteriophage was determined by plaque assay and expressed as plaque-forming units per milliliter (PFU/mL).

Serial dilutions of the purified phage preparations were prepared, and suitable dilutions were subjected to plaque assay. After overnight incubation at 37°C, discrete plaques were counted, and the phage titer was calculated from the number of plaques, dilution factor, and volume plated. For each phage, the dilution reported was the highest dilution yielding discrete, non-confluent plaques that could be counted; lower dilutions produced confluent lysis and were not countable. Titration was performed once per phage, and replicate titrations were not carried out.

The titer was calculated using the following formula:

$\text{Titer (PFU/mL)} = \frac{\text{Number of plaques} \times \text{Dilution factor}}{\text{Volume plated (mL)}}$

The estimated titers obtained were 1.6 × 10¹² PFU/mL for Phage-1, 1.7 × 10¹⁶ PFU/mL for Phage-2, and 1.9 × 10¹⁵ PFU/mL for Phage-3.

Host range and lytic activity assessment

The host range of the three purified bacteriophages was evaluated against all 375 MDR *E. coli *clinical isolates. Each isolate was tested separately against Phage-1, Phage-2, and Phage-3 using the spot assay. A clearly demarcated zone of lysis at the site of phage application was recorded as a positive lytic reaction. The lytic nature of the three phages had already been established by plaque formation during their isolation and purification; however, individual isolate-phage combinations in the host-range screening were not separately re-confirmed by plaque assay or efficiency-of-plating (EOP) testing. The host-range results therefore represent phenotypic susceptibility to spot-test lysis. The proportion of isolates showing detectable lysis was calculated for each individual phage. The observed lytic activity was 146 (38.9%) isolates for Phage-1, 90 (24.0%) isolates for Phage-2, and 169 (45.1%) isolates for Phage-3.

Phage susceptibility pattern

The results obtained from testing the same 375 MDR *E. coli *isolates against each of the three individual phages were used to classify the isolates according to their susceptibility patterns. The following susceptibility categories were recorded: Phage-1 only, Phage-2 only, Phage-3 only, Phage-1 + Phage-2, Phage-1 + Phage-3, Phage-2 + Phage-3, all three phages, and no detectable lysis with any of the three phages. An isolate showing detectable lysis with at least one individual phage was considered susceptible to at least one phage. These categories represented isolate-level susceptibility patterns and were not interpreted as evidence of synergistic interaction between the phages.

Cumulative coverage of the three phages

The combined coverage of Phage-1, Phage-2, and Phage-3 was derived from the individual host-range results obtained for the same 375 MDR *E. coli *clinical isolates. An isolate was counted as covered if it showed detectable lysis with at least one of the three individual phages. No physically mixed phage cocktail was prepared or tested separately against the isolates. The number and percentage of isolates covered by at least one of the three phages were recorded. The cumulative coverage of the three phages was 260 (69.3%) isolates, while 115/375 (30.7%) isolates showed no detectable lysis with any of the three phages.

This cumulative figure reflects the union of the individual host-range results and was not interpreted as evidence of synergistic interaction, as neither a physically mixed cocktail nor a dedicated quantitative synergy assay was evaluated.

Statistical analysis

Because the same 375 MDR *E. coli *isolates were tested against all three individual phages, the observations were paired. The overall difference in lytic activity among Phage-1, Phage-2, and Phage-3 was evaluated using Cochran's Q test for related binary observations. Pairwise comparisons were subsequently performed using exact McNemar tests, with Bonferroni correction for multiple comparisons. All statistical analyses were performed using IBM SPSS Statistics, version 29.0 (IBM Corp., Armonk, NY). A p-value of <0.05 was considered statistically significant.

Ethical considerations

The study involved clinical bacterial isolates obtained during routine diagnostic microbiological investigations. Patient-identifying information was not used in the laboratory analysis or presentation of the results. This study did not involve human participants. The study protocol was approved by the Institutional Ethics Committee of Santosh Deemed to be University, Ghaziabad, India (F. No. SU/R/2024/2403(3), dated 05.09.2024), and the Institutional Ethics Committee of Heritage Institute of Medical Sciences, Varanasi, India (Ref. No. HIMS/IEC/317, dated 15.09.2025). All clinical bacterial isolates and environmental samples were handled in accordance with the World Health Organization Laboratory Biosafety Manual [10].

## Results

Clinical characteristics of MDR *Escherichia coli *isolates

A total of 375 multidrug-resistant (MDR) *E. coli *isolates recovered from clinical specimens were included in the study. Urine was the predominant clinical specimen, accounting for 240 (64.0%) isolates, followed by pus, 65 (17.3%), sputum, 26 (6.9%), blood, 20 (5.3%), tissue, 9 (2.4%), Foley catheter tips, 6 (1.6%), high vaginal swabs, 6 (1.6%), and endotracheal tube specimens, 3 (0.8%) (Table 1).

**Table 1:** Clinical specimen distribution of MDR E. coli isolates MDR, multidrug-resistant

Clinical specimen	Number (n)	Percentage (%)
Urine	240	64.0
Pus	65	17.3
Sputum	26	6.9
Blood	20	5.3
Tissue	9	2.4
Foley catheter tip	6	1.6
High vaginal swab	6	1.6
Endotracheal tube	3	0.8
Total	375	100.0

Antimicrobial susceptibility profile

The antimicrobial susceptibility pattern of the 375 MDR *E. coli* isolates is presented in Table 2. The highest level of resistance was observed against ciprofloxacin (357; 95.2%), followed by ceftriaxone (319; 85.1%), trimethoprim-sulfamethoxazole (280; 74.7%), amoxicillin-clavulanic acid (278; 74.1%), and piperacillin-tazobactam (270; 72.0%). Resistance to cefepime and cefuroxime was observed in 251 (66.9%) and 234 (62.4%) isolates, respectively, as shown in Figure 1. Among the tested antibiotics, the highest susceptibility was observed to amikacin (271; 72.3%), followed by gentamicin (221; 58.9%). Among the 240 urinary isolates tested with norfloxacin, fosfomycin, and nitrofurantoin, susceptibility was 39.6%, 62.1%, and 37.9%, respectively.

**Table 2:** Antimicrobial susceptibility pattern of MDR E. coli isolates *Tested in 240 urinary isolates. MDR, multidrug-resistant

Antibiotic	Tested	R (%)	I (%)	S (%)
Ampicillin	375	159 (42.4)	0 (0.0)	216 (57.6)
Amoxicillin-clavulanic acid	375	278 (74.1)	28 (7.5)	69 (18.4)
Piperacillin-tazobactam	375	270 (72.0)	0 (0.0)	105 (28.0)
Cefuroxime	375	234 (62.4)	0 (0.0)	141 (37.6)
Ceftriaxone	375	319 (85.1)	0 (0.0)	56 (14.9)
Ceftazidime	375	188 (50.1)	8 (2.1)	179 (47.7)
Cefoperazone-sulbactam	375	225 (60.0)	3 (0.8)	147 (39.2)
Cefepime	375	251 (66.9)	20 (5.3)	104 (27.7)
Ertapenem	375	205 (54.7)	9 (2.4)	161 (42.9)
Imipenem	375	194 (51.7)	8 (2.1)	173 (46.1)
Meropenem	375	207 (55.2)	4 (1.1)	164 (43.7)
Amikacin	375	101 (26.9)	3 (0.8)	271 (72.3)
Gentamicin	375	148 (39.5)	6 (1.6)	221 (58.9)
Ciprofloxacin	375	357 (95.2)	9 (2.4)	9 (2.4)
Norfloxacin*	240	117 (48.8)	28 (11.7)	95 (39.6)
Ofloxacin	375	197 (52.5)	5 (1.3)	173 (46.1)
Fosfomycin*	240	80 (33.3)	11 (4.6)	149 (62.1)
Trimethoprim-sulfamethoxazole	375	280 (74.7)	0 (0.0)	95 (25.3)
Nitrofurantoin*	240	111 (46.3)	38 (15.8)	91 (37.9)

**Figure 1:**
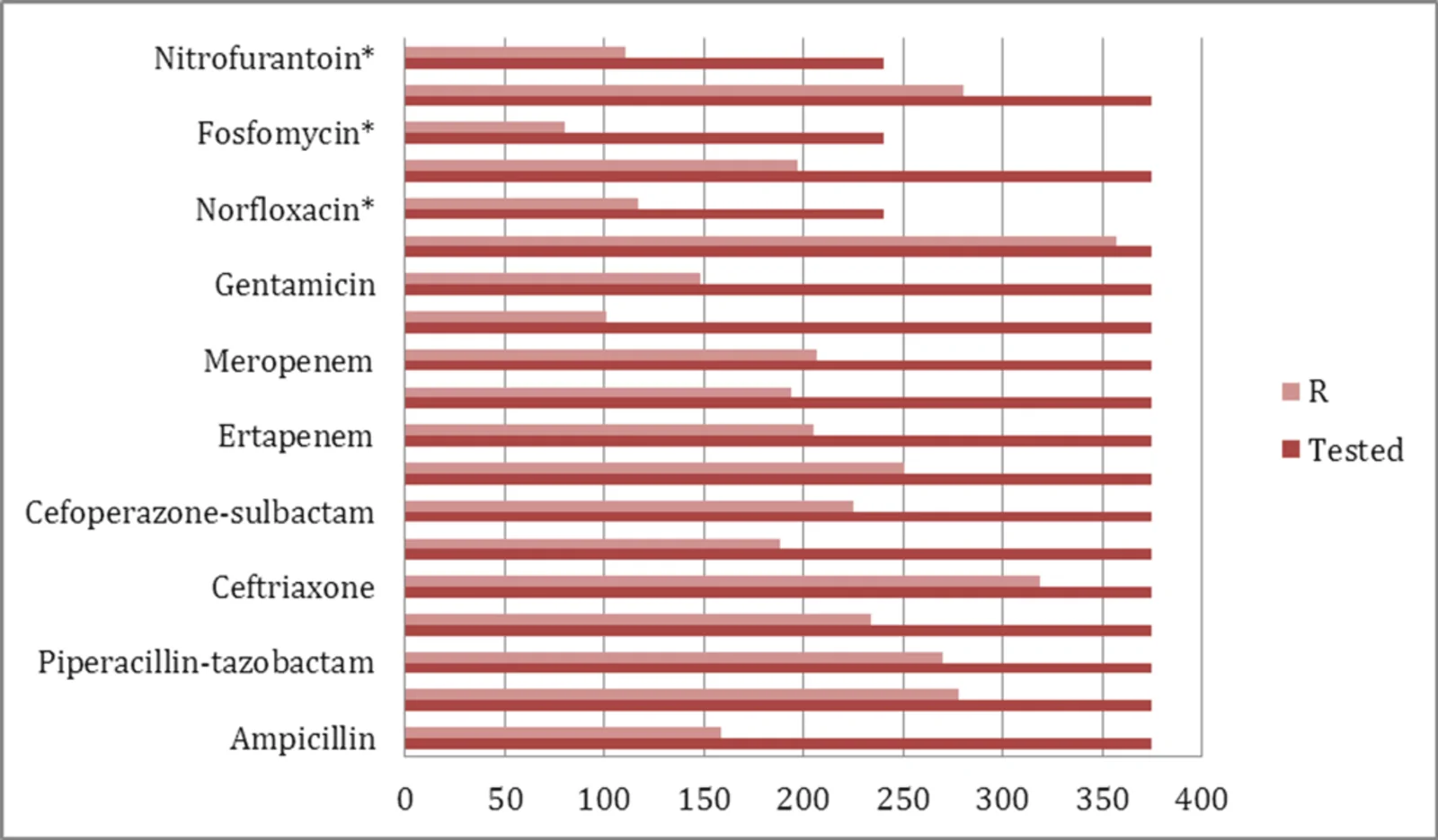
Bar graph showing resistance (%) to the major antibiotics. *Norfloxacin, fosfomycin, and nitrofurantoin were tested only in the 240 urinary isolates; all other antibiotics were tested in all 375 isolates.

Isolation and preliminary screening of bacteriophages

Three environmental water sources - pond water, sewage water, and Ganga water - were screened for bacteriophages active against MDR *E. coli*. One sample was collected from each source.

Of the 375 MDR *E. coli* isolates screened, 72 (19.2%) showed a positive reaction in the preliminary spot assay (Table 3). Following confirmation by plaque assay, 36 (9.6%) isolates showed plaque formation, while three purified bacteriophage isolates (0.8%) were ultimately recovered; representative images of the spot assay and plaque assay are shown in Figures 2, 3, respectively. The three purified phages were designated Phage-1 (P1), Phage-2 (P2), and Phage-3 (P3), originating from pond water, sewage water, and Ganga water, respectively.

**Table 3:** Isolation of bacteriophages from environmental water sources

Screening stage	Number (N)	Percentage (%)
MDR *E. coli *isolates screened	375	100.0
Spot assay positive	72	19.2
Plaque assay positive	36	9.6
Purified phage isolates	3	0.8

**Figure 2:**
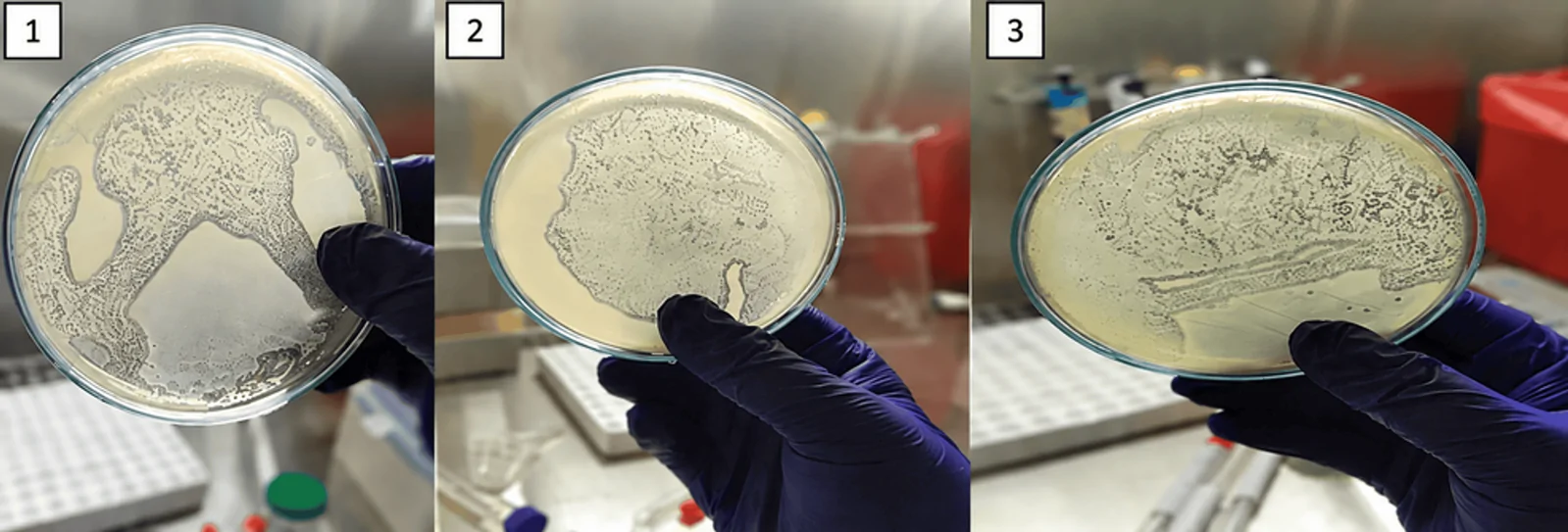
Representative spot assay showing zones of lysis. (1-3) Spot assay plates for Phage-1, Phage-2, and Phage-3, respectively, against susceptible host isolates; zones of clearing indicate phage-mediated lysis. The photographs are original and were taken by the authors in their laboratory.

**Figure 3:**
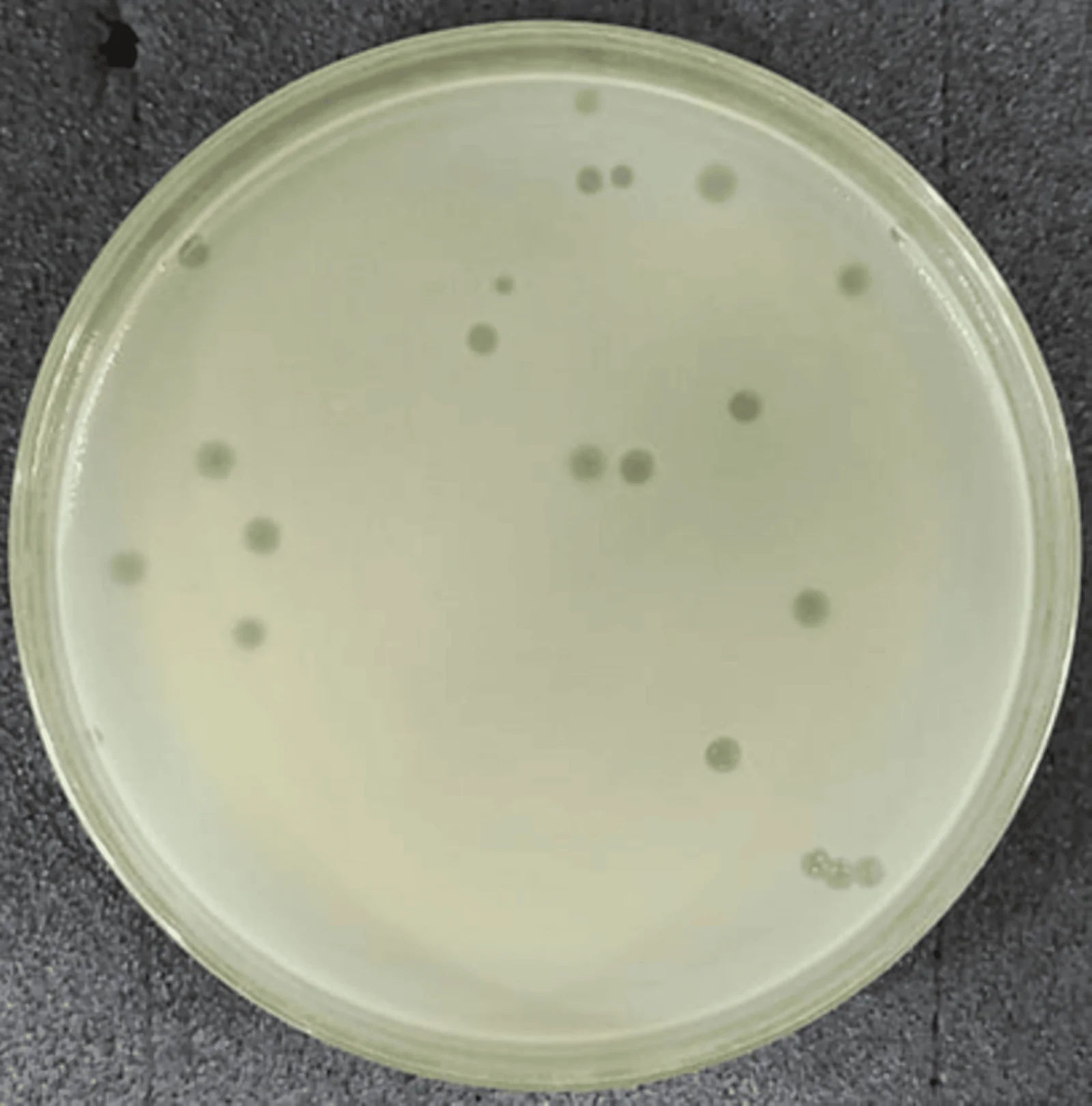
Representative double-layer agar plaque assay showing discrete plaques. The photograph is original and was taken by the authors in their laboratory.

Phage titer

Following plaque purification and propagation, the titers of the three purified bacteriophages were determined by plaque assay and expressed as plaque-forming units per milliliter (PFU/mL). Phage-1 (P1), isolated from pond water, showed an estimated titer of 1.6 × 10¹² PFU/mL at a dilution of 10⁻¹¹, based on approximately 16 plaques. Phage-2 (P2), recovered from sewage water, showed the highest estimated titer of 1.7 × 10¹⁶ PFU/mL at a dilution of 10⁻¹⁵, based on approximately 17 plaques. Phage-3 (P3), isolated from Ganga water, showed an estimated titer of 1.9 × 10¹⁵ PFU/mL at a dilution of 10⁻¹³, based on approximately 190 plaques (Table 4). The plaque counts for Phage-1 and Phage-2 (16 and 17 plaques, respectively) were below the conventional countable range of 30-300 plaques per plate, and the corresponding titers should therefore be regarded as approximate estimates.

**Table 4:** Phage titer of purified bacteriophage isolates

Phage	Source	Dilution	Approx. plaques	Volume	Estimated titer (PFU/mL)
Phage-1	Pond water	10⁻¹¹	16	1.0 mL	1.6 × 10¹²
Phage-2	Sewage water	10⁻¹⁵	17	1.0 mL	1.7 × 10¹⁶
Phage-3	Ganga water	10⁻¹³	~190	1.0 mL	1.9 × 10¹⁵

Host range and lytic activity of purified bacteriophages

The host range of the three purified bacteriophages was evaluated against all 375 MDR *E. coli *clinical isolates using the spot assay. Lytic activity was observed for all three phages, although the proportion of susceptible isolates varied between phages.

Phage-3 demonstrated the highest observed lytic activity, with lysis detected in 169 of 375 isolates (45.1%; 95% CI: 40.1-50.1%). Phage-1 produced lysis in 146 isolates (38.9%; 95% CI: 34.1-44.0%), whereas Phage-2 showed lytic activity against 90 isolates (24.0%; 95% CI: 20.0-28.6%). The corresponding numbers of isolates without detectable lysis were 206 (54.9%), 229 (61.1%), and 285 (76.0%) for P3, P1, and P2, respectively, as summarized in Table 5.

**Table 5:** Lytic activity of individual bacteriophages against 375 MDR E. coli isolates MDR, multidrug-resistant

Phage	Tested	Lysis positive, n (%)	95% CI	Lysis negative, n (%)
Phage-1	375	146 (38.9)	34.1–44.0	229 (61.1)
Phage-2	375	90 (24.0)	20.0–28.6	285 (76.0)
Phage-3	375	169 (45.1)	40.1–50.1	206 (54.9)

Phage susceptibility patterns

Among the 375 MDR *E. coli* isolates, 51 (13.6%) were susceptible to Phage-1 only, 20 (5.3%) to Phage-2 only, and 84 (22.4%) to Phage-3 only. Combined susceptibility to Phage-1 and Phage-2 was observed in 20 (5.3%) isolates, while 35 (9.3%) were susceptible to Phage-1 and Phage-3 and 10 (2.7%) to Phage-2 and Phage-3. A total of 40 (10.7%) isolates were susceptible to all three phages. Overall, 115 (30.7%) isolates showed no detectable lysis by any of the three individual phages. Thus, susceptibility to at least one of the three phages was observed in 260/375 (69.3%) isolates (Table 6).

**Table 6:** Phage susceptibility patterns among MDR E. coli isolates Note: These categories represent isolate-level susceptibility patterns to individual phages and do not by themselves demonstrate synergistic interaction between phages. MDR, multidrug-resistant

Pattern	Number (n)	Percentage (%)
Phage-1 only	51	13.6
Phage-2 only	20	5.3
Phage-3 only	84	22.4
Phage-1 + Phage-2	20	5.3
Phage-1 + Phage-3	35	9.3
Phage-2 + Phage-3	10	2.7
All three phages	40	10.7
No detectable lysis with any of the three phages	115	30.7
Total	375	100.0

Statistical comparison of lytic activity

Because the same 375 MDR *E. coli *isolates were tested against all three individual phages, a repeated-measures comparison was performed using Cochran's Q test. The overall comparison demonstrated a statistically significant difference in lytic activity among the three phages (Cochran's Q = 45.03, df = 2, p < 0.001).

Pairwise comparisons were subsequently performed using exact McNemar tests with Bonferroni correction. A statistically significant difference was observed between Phage-1 and Phage-2 (Bonferroni-adjusted p < 0.001) and between Phage-2 and Phage-3 (Bonferroni-adjusted p < 0.001). In contrast, the difference between Phage-1 and Phage-3 was not statistically significant (Bonferroni-adjusted p = 0.259). Although Phage-3 showed the highest observed lytic activity (45.1%) and Phage-1 showed 38.9%, their difference was not statistically significant. Both Phage-1 and Phage-3 demonstrated significantly greater lytic activity than Phage-2 (24.0%), as detailed in Table 7.

**Table 7:** Statistical comparison of lytic activity among individual phages Overall test: Cochran's Q = 45.03; df = 2; p < 0.001.

Comparison	Test	Bonferroni-adjusted p-value	Interpretation
Phage-1 vs Phage-2	Exact McNemar	<0.001	Significant
Phage-1 vs Phage-3	Exact McNemar	0.259	Not significant
Phage-2 vs Phage-3	Exact McNemar	<0.001	Significant

Cumulative coverage of the three phages

The combined coverage of Phage-1, Phage-2, and Phage-3 was derived from the individual host-range results for the same 375 MDR *E. coli *isolates. Isolates showing detectable lysis with at least one of the three phages numbered 260 (69.3%), while 115 (30.7%) showed no detectable lysis with any phage.

This cumulative coverage was higher than the individual lytic activity of Phage-1 (38.9%), Phage-2 (24.0%), or Phage-3 (45.1%), as compared in Table 8, and reflects the complementary susceptibility patterns observed among the individual phages. Because the figure represents the union of individual host-range results rather than a separately tested phage mixture, it is reported as cumulative coverage and not as evidence of synergistic interaction between the phages. This is a descriptive comparison only; no statistical test was applied between the cumulative coverage and the activity of the individual phages, as the cumulative figure is derived from the same individual observations and is not an independent measurement.

**Table 8:** Comparative activity of individual phages and their cumulative coverage

Treatment	Tested	Lysis positive, n (%)	Lysis negative, n (%)
Phage-1	375	146 (38.9)	229 (61.1)
Phage-2	375	90 (24.0)	285 (76.0)
Phage-3	375	169 (45.1)	206 (54.9)
Three phages (cumulative coverage)	375	260 (69.3)	115 (30.7)

## Discussion

The present study evaluated the isolation and in vitro lytic activity of bacteriophages against 375 MDR *E. coli *clinical isolates. The study demonstrated a high level of AMR among the clinical isolates and identified three bacteriophages from environmental water sources with variable lytic activity against the study isolates. Phage-1, Phage-2, and Phage-3 showed lysis in 38.9%, 24.0%, and 45.1% of isolates, respectively, and their cumulative coverage was 69.3%. These findings demonstrate variation in the activity of individual phages and broader coverage when their host ranges are considered together.

AMR pattern

In the present study, the isolates showed the highest resistance to ciprofloxacin among the tested antimicrobial agents, with 357/375 (95.2%) isolates resistant. This was followed by ceftriaxone (85.1%), trimethoprim-sulfamethoxazole (74.7%), amoxicillin-clavulanic acid (74.1%), and piperacillin-tazobactam (72.0%). Resistance was also substantial to cefepime (66.9%), cefuroxime (62.4%), cefoperazone-sulbactam (60.0%), meropenem (55.2%), ertapenem (54.7%), and imipenem (51.7%) [1].

The high level of fluoroquinolone resistance observed in the present study is consistent with the substantial fluoroquinolone resistance reported among clinical and urinary *E. coli* isolates from different settings. However, direct comparison of percentages should be made cautiously because AMR varies according to geographical location, patient population, specimen type, healthcare setting, and selection criteria. In the present study, all 375 isolates were already selected as MDR isolates; therefore, the resistance levels are expected to be higher than those reported in studies examining unselected or community-acquired *E. coli* populations [1].

The high resistance to ceftriaxone and other cephalosporins observed in the present study further demonstrates the extensive resistance burden within this selected MDR population. However, the present study did not investigate the molecular mechanisms responsible for these resistance phenotypes. Therefore, the observed resistance should not be attributed specifically to ESBL, AmpC, or carbapenemase production without corresponding phenotypic or molecular testing.

Among the antimicrobials tested in all 375 isolates, the isolates showed the highest susceptibility to amikacin, with 271 (72.3%) isolates susceptible, followed by gentamicin, with 221 (58.9%) susceptible isolates. The relatively greater activity of amikacin in the present study indicates that some conventional antimicrobial options retained activity against a proportion of the MDR isolates. Nevertheless, the overall resistance profile demonstrates the substantial AMR burden of the study population and provides an important clinical context for investigating alternative antibacterial approaches.

Isolation of bacteriophages from environmental sources

Three environmental water sources - pond water, sewage water, and Ganga water - were screened for bacteriophages active against MDR *E. coli*. Preliminary spot screening produced positive reactions in 72 (19.2%) of the 375 isolates, while plaque formation was observed in 36 (9.6%) isolates. Three purified bacteriophages were subsequently recovered and designated Phage-1, Phage-2, and Phage-3 according to their environmental sources.

The recovery of phages from these environmental sources is consistent with the established ecological distribution of bacteriophages. Phages infecting *E. coli* have been isolated from diverse natural environments, including ponds, rivers, wastewater, sewage, and other environments containing their bacterial hosts. A broad *E. coli *phage collection study demonstrated that environmental samples can serve as useful reservoirs of phages with different host ranges. Similarly, Vera-Mansilla et al. isolated and characterized lytic phages against MDR clinical *E. coli*, supporting the feasibility of obtaining clinically relevant phages from environmental sources [2,6,12].

In the present study, spot assay was followed by plaque assay and plaque purification. This sequential approach allowed preliminary detection of phage-associated lysis followed by recovery of purified phage isolates capable of producing plaques under the assay conditions. The reduction from 72 spot-positive reactions to 36 plaque-positive isolates indicates that fewer isolates demonstrated plaque formation under the subsequent assay conditions. The present study does not, however, establish the specific reason for this difference [12].

Phage titer

Following propagation and plaque assay, all three purified phages demonstrated high estimated titers. Phage-1 showed a titer of 1.6 × 10¹² PFU/mL, Phage-2 showed a titer of 1.7 × 10¹⁶ PFU/mL, and Phage-3 showed a titer of 1.9 × 10¹⁵ PFU/mL.

The difference in titer among the three phages demonstrates variation in their propagation characteristics under the experimental conditions used. Phage titer is influenced by several biological and experimental factors, including the interaction between the phage and its propagation host and the efficiency of phage replication and release. Therefore, the higher titer observed for Phage-2 should not by itself be interpreted as indicating a broader host range or greater lytic activity. This is supported by the present findings, in which Phage-2 had the highest estimated titer but the lowest observed lytic coverage among the three phages [6,12].

Individual phage lytic activity

The host-range analysis demonstrated considerable variation in the lytic activity of the three purified phages. Phage-1 lysed 146/375 (38.9%) isolates, Phage-2 lysed 90/375 (24.0%) isolates, and Phage-3 lysed 169/375 (45.1%) isolates. Phage-3 therefore demonstrated the highest observed lytic activity, whereas Phage-2 demonstrated the lowest observed lytic activity.

Variable host range among *E. coli *phages has been reported previously. Vera-Mansilla et al. characterized three lytic phages against MDR clinical *E. coli *and demonstrated differences in phage activity among bacterial isolates. Similarly, Maffei et al., using a large collection of *E. coli* phages, demonstrated substantial variation in phage-host interactions and showed that host recognition and bacterial defense mechanisms contribute to differences in phage susceptibility [2,4,12].

The variable lytic activity observed in the present study may therefore reflect differences in phage-host compatibility among the clinical isolates. Phage attachment requires recognition of appropriate bacterial surface structures, and differences in bacterial receptors and defense mechanisms can influence whether productive infection occurs. However, because receptor characterization and genomic analysis are outside the scope of the present analysis, the specific mechanism responsible for susceptibility or resistance of individual isolates cannot be established from these phenotypic results alone [4,12].

The finding that Phage-3 showed the highest observed activity should also be interpreted carefully. Although Phage-3 lysed 45.1% of isolates compared with 38.9% for Phage-1, the statistical comparison between these two phages was not significant. Therefore, the observed numerical difference does not establish statistically greater activity of Phage-3 over Phage-1.

Statistical comparison of individual phages

Because the same 375 MDR *E. coli *isolates were tested against all three individual phages, the observations were paired. Cochran's Q test demonstrated a statistically significant overall difference in lytic activity among the three phages (Q = 45.03, df = 2, p < 0.001).

Pairwise exact McNemar testing with Bonferroni correction demonstrated significant differences between Phage-1 and Phage-2 and between Phage-2 and Phage-3. In contrast, the difference between Phage-1 and Phage-3 was not statistically significant (adjusted p = 0.259).

Thus, the overall significant result was mainly associated with the lower lytic activity of Phage-2 compared with Phage-1 and Phage-3. Although Phage-3 had the highest observed lysis percentage, the statistical analysis does not support describing Phage-3 as significantly superior to Phage-1. This distinction is important because an observed difference in percentages should not be interpreted as a statistically significant difference without an appropriate paired comparison.

Cumulative coverage of the three phages

The cumulative coverage of Phage-1, Phage-2, and Phage-3, derived from the individual host-range results for the same collection of 375 MDR *E. coli *isolates, was 260/375 (69.3%), whereas 115 (30.7%) isolates showed no detectable lysis with any of the three phages.

The 69.3% cumulative coverage was higher than the activity of each individual phage: Phage-1, 38.9%; Phage-2, 24.0%; and Phage-3, 45.1%. The absolute difference between the cumulative coverage and Phage-3 alone was 24.2 percentage points.

This finding is consistent with previous studies demonstrating that combining phages with different or overlapping host ranges can increase the proportion of bacterial isolates susceptible to phage treatment. An open-access study evaluating six *E. coli* phages reported overlapping but different host ranges among individual phages and showed that the phages collectively interacted with a greater proportion of the tested bacterial collection than most individual phages. Another study investigating a broad-host-range phage cocktail against antimicrobial-resistant *E. coli *emphasized that the narrow host range of individual phages is an important limitation and that combining phages can increase the range of susceptible bacterial hosts [5,7,13].

Similarly, an open-access study of environmental phages against clinical *E. coli *demonstrated that cocktails composed of a small number of individual phages could substantially increase coverage compared with individual phages. These findings provide a rationale for evaluating phage combinations where complementary host ranges are observed, as in the present study [5,7,13].

However, the present finding should be interpreted specifically as cumulative host-range coverage and not as evidence of synergistic interaction. A physically mixed phage cocktail was not prepared or tested separately, and no dedicated quantitative synergy experiment was performed. The present data therefore indicate that 69.3% of isolates were susceptible to at least one of the three phages, but they do not establish the performance of a formulated cocktail or any pharmacological or biological synergy between Phage-1, Phage-2, and Phage-3.

It is also important to distinguish Phage-3 alone from the cumulative coverage of the three phages. Phage-3 alone produced lysis in 169/375 (45.1%) isolates, whereas 260/375 (69.3%) isolates were susceptible to at least one of the three phages. The latter figure is a derived cumulative value and does not represent a separate phage treatment.

Overall interpretation

Overall, the present study demonstrated that environmental water sources yielded three bacteriophages capable of producing detectable lysis against MDR *E. coli* clinical isolates. The individual phages showed different levels of lytic activity, with Phage-3 showing the highest observed activity and Phage-2 the lowest observed activity. Statistical analysis demonstrated a significant overall difference among the three phages, while the difference between Phage-1 and Phage-3 was not statistically significant.

The cumulative coverage of the three phages was broader than that of any individual phage, with 69.3% of the tested MDR *E. coli* isolates susceptible to at least one phage. This finding supports the concept that combining phages with complementary activity may increase the breadth of bacterial coverage. Nevertheless, the result represents a derived in vitro phenotypic observation and should not be interpreted as evidence of cocktail performance, clinical efficacy, or phage synergy.

Further characterization of these phages, including detailed biological and genomic evaluation, would be necessary to assess their suitability for subsequent phage-development studies. The present findings therefore provide a phenotypic basis for further investigation of these environmental bacteriophages against MDR *E. coli* [3,6,12,14].

Limitations

This study has several limitations. Only one environmental sample was collected from each water source, and thus the findings may not reflect the full phage diversity present at these sites or at other times. A standard reference strain such as *E. coli *ATCC 25922 was not included as a screening or propagation host, as the study focused on phage activity against contemporary clinical MDR isolates. Host-range positivity was defined by spot-test clearing, which may, in some instances, reflect lysis from without rather than productive infection. Positive reactions were not individually confirmed by plaque formation or efficiency-of-plating assays for each isolate-phage combination, and the reported coverage should therefore be interpreted as phenotypic lysis rather than confirmed productive infection. Phage titers were derived from a single countable plate per phage without replicate titrations, and the plaque counts for Phage-1 and Phage-2 fell below the conventional countable range; thus, the reported titers are approximate, and their precision is limited. A physically mixed three-phage cocktail was not prepared or tested separately against the isolates; the 69.3% figure represents cumulative coverage derived from the individual host-range results, and neither the performance of a formulated cocktail nor any synergistic interaction between the phages was evaluated, as no dedicated quantitative synergy assay was undertaken. Phage characterization was limited to phenotypic assays: genomic sequencing and receptor identification were not performed, and thus the taxonomic identity of the phages, their lytic versus temperate lifecycle, the absence of undesirable genetic determinants such as lysogeny-associated, AMR, or virulence genes, and the molecular basis of the observed lytic activity all remain unconfirmed; such characterization is essential before any of these phages could be considered for therapeutic development. Host-range testing against non-MDR isolates was not undertaken, and AMR mechanisms (e.g., ESBL, AmpC, or carbapenemase production) were not confirmed phenotypically or molecularly. Finally, all experiments were conducted under in vitro laboratory conditions, and the findings do not establish clinical efficacy or safety; in vivo and translational studies are required before these phages can be considered for therapeutic application.

## Conclusions

This study shows that environmental water sources can serve as a practical reservoir for bacteriophages with lytic activity against MDR *E. coli*. Individual phages isolated from these sources varied considerably in their host range, producing detectable lysis in 146/375 (38.9%) isolates for Phage-1, 90/375 (24.0%) for Phage-2, and 169/375 (45.1%) for Phage-3, which underscores the value of screening multiple candidate phages before selecting agents for further development. Considered together, the three phages covered 260/375 (69.3%) isolates, a larger proportion than any single phage, supporting the rationale for evaluating cocktail-based strategies against genetically diverse clinical populations. This figure represents cumulative in vitro phenotypic coverage derived from the individual host-range results, not the performance of a formulated cocktail or any measure of therapeutic effectiveness.

These findings should be regarded as a preliminary in vitro phenotypic characterization rather than evidence of therapeutic efficacy or phage synergy. Genomic characterization to establish phage taxonomic identity, confirm a strictly lytic lifecycle, and exclude lysogeny-associated, AMR, and virulence genes, together with receptor identification and dedicated synergy and safety testing, is needed before these bacteriophages can be considered candidates for translational or clinical phage-therapy applications.
